# Lentinan suppresses the progression of neuroblastoma by inhibiting FOS-mediated transcription activation of VRK1 to stabilize p53 protein

**DOI:** 10.1038/s41420-025-02315-0

**Published:** 2025-03-15

**Authors:** Zhang Zhao, Jiahao Li, Liyu Zhang, Jiayu Wang, Dian Li, Manna Zheng, Zijie Ye, Tianyou Yang, Yan Zou, Jing Pan, Hui Xu, Huijuan Zeng, Chao Hu

**Affiliations:** 1 Department of Pediatric Surgery, Guangzhou Institute of Pediatrics, Guangzhou Women and Children’s Medical Center, Guangzhou Medical University, Guangdong Provincial Clinical Research Center for Child Health, Guangzhou, 510623 China; 2 Department of Radiology, Nanjing First Hospital, Nanjing Medical University, 210006 Nanjing, Jiangsu China

**Keywords:** Neurological disorders, Neuroscience

## Abstract

Neuroblastoma (NB) is a common malignant and solid pediatric tumor with unfavorable prognosis. Although studies have shown the anti-tumor efficacy of lentinan (LNT), molecular mechanism that contribute to the anti-tumor effect on NB remains unclear. The aim of this study is to unmask the anti-tumor role of LNT in NB and the specific molecular mechanism. At first, the in vivo experiments were conducted and the results indicated that LNT could suppress tumor growth in NB. Subsequent cellular functional assays unveiled that LNT treatment could efficiently decrease NB cell viability, induce cell cycle stagnation at G0/G1 phase, increase the apoptosis rate, and weaken the migrating and invasive abilities. Furthermore, LNT resulted in a significant downregulation of FOS expression. FOS overexpression recovered the growth, migration and invasion of NB cells suppressed by LNT treatment. Mechanism investigations revealed that FOS interacted with JUND to transcriptionally activate VRK1. Moreover, VRK1 downregulated p53 protein via inducing the phosphorylation of p53 at site 291–393. In summary, this study reveals a novel molecular pathway by which LNT exerts tumor-suppressing functions in NB.

## Introduction

Neuroblastoma (NB) is one of the commonest extracranial solid tumors in children, which derives from embryonic neural crest cells [1]. It is characterized by the rapid growth and early metastasis, thus accounting for 10–15% of all malignant tumor-related death in children [2]. Therefore, it is urgent to find new therapeutic targets and effective drugs. Lentinan (LNT) is an active extract isolated from the shiitake mushroom. Studies have validated multiple functions of LNT, including antioxidant, immunomodulatory, anti-tumor, hypoglycemic, and hypolipidemic functions [3–5]. LNT is a kind of natural macromolecule with a β-1,3-D-glucan, and its unique structure and component are crucial for its anti-tumor function [6, 7]. As reported, LNT has been used for adjuvant tumor therapy in China [8]. However, whether LNT has tumor-suppressing function in NB remains unknown. Our current study aims to unmask the specific role of LNT in regulating NB progression.

Fos proto-oncogene, AP-1 transcription factor subunit (FOS) gene has been recognized as an oncogene in human malignant tumors [9–11], including NB [12, 13]; moreover, it can be modulated in cancer cells treated with anti-cancer drugs [14, 15]. It is unreported whether LNT can regulate the expression of the oncogenic FOS gene to affect NB progression. The current study focuses on the role of LNT-mediated FOS expression changes in modulating NB progression.

p53 is a tumor suppressor playing a vital role in regulating cancer cell cycle, apoptosis, senescence, and DNA repair [16]. p53 can directly interact with MDM2 that can downregulate p53 to suppress its anti-tumor functions [17]. p53-MDM2 pathway is a crucial factor regulating cell apoptosis and cell cycle [18]. Evidence has revealed the suppressing effect of FOS on p53 [19]. However, the specific mechanism of FOS-mediated p53 downregulation in NB progression remains to be explored. Mechanistically, FOS can act as a transcription regulator to activate the transcription of its target genes. For example, FOS can induce NANOG transcription to promote 5-FU resistance in colon cancer [20]. c-Fos/c-Jun-mediated transcription activation of poFUT1 promotes embryo adhesion [21]. FOS can cooperate with Jun to exert the function of transcriptional regulation [22]. In this study, we explored the effect of FOS-mediated transcriptional regulation on p53 expression.

Vaccinia-related kinase 1 (VRK1) is a nuclear kinase that is pro-proliferative. A large number of reports have unveiled the close correlation between the upregulation of VRK1 and the malignant progression of human cancers [23, 24]. Based on the bioinformatics analysis, we made further investigation on the FOS-induced transcriptional activation of VRK1 in NB cells. Moreover, studies have indicated that VRK1 and p53 have interlinks and can affect each other [25, 26]. Considering the regulating effects of FOS on VRK1 and p53, we further explored the specific regulating mechanism by which VRK1 affects p53 protein expression.

In summary, the focuses of this study are to unmask the anti-tumor efficacy of LNT in NB and to explore whether LNT-mediated inhibition of FOS expression can promote the upregulation of the tumor suppressor p53.

## Results

### LNT suppresses NB growth in in vivo tumor models

**Fig. 1:**
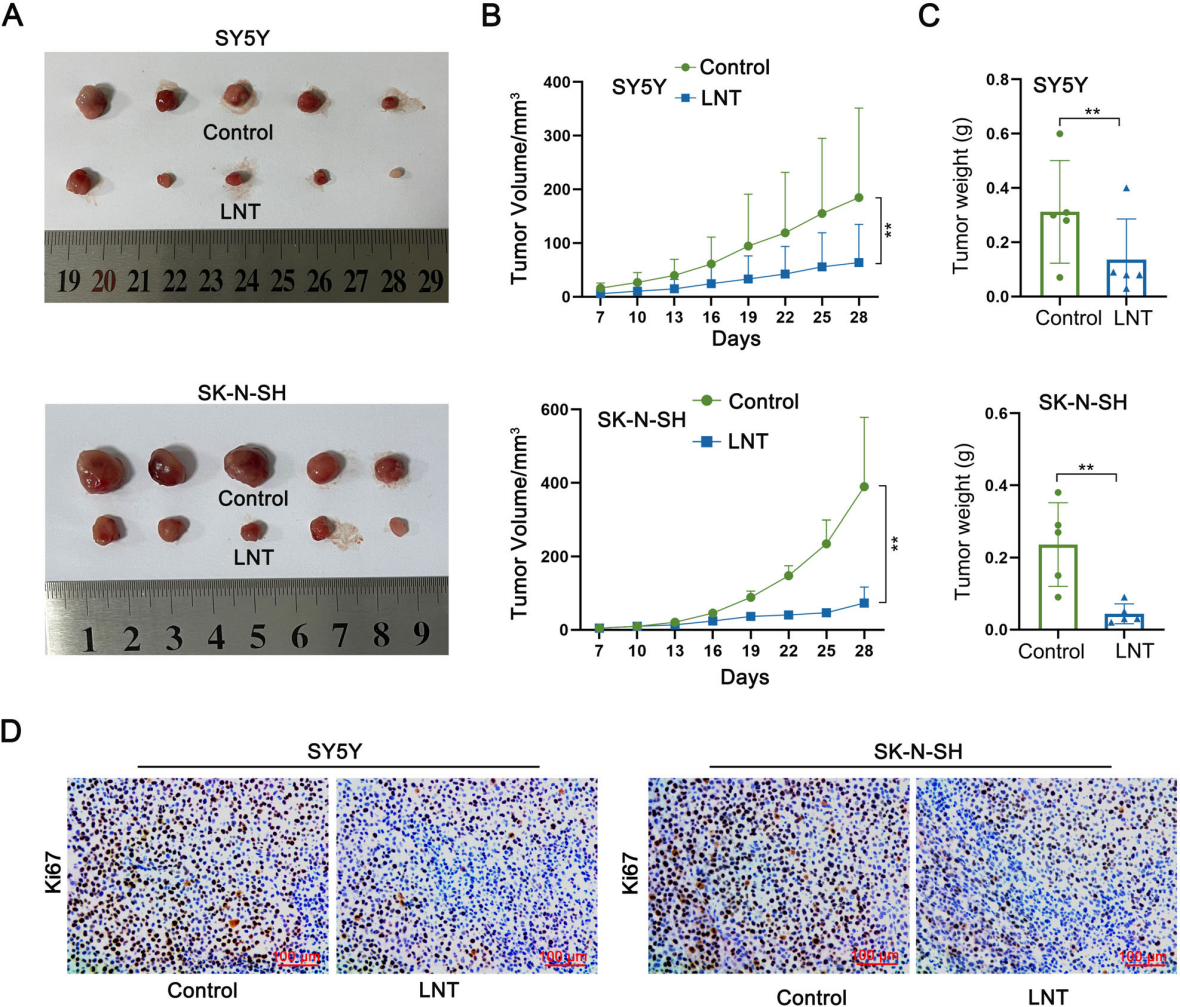
LNT suppresses NB growth in in vivo tumor models. **A** Tumor-bearing model mice was injected with PBS or LNT. Tumors were resected for size measurement on 28th day after injection. **B** Tumor growth curves of two groups of mice were plotted to show the difference of tumor volume. **C** The weight of tumors in LNT group and Control group was measured. **D** The positive expression of proliferation marker (ki67) in tumor tissues isolated from two groups of tumors was detected by IHC. **P < 0.01.

The efficacy of LNT in altering NB tumor growth was unmasked through establishing tumor-bearing model mice and then injected them with PBS or LNT to make in vivo investigations. The results showed that the tumor size in LNT group was smaller than that in Control group (Fig. 1A). Moreover, the tumor growth rate was slowed down in mice treated with LNT, as evidenced by the smaller tumor volume (Fig. 1B). Through further measurement, the weight of tumors in LNT group was lighter than that in Control group (Fig. 1C). In addition, IHC data unmasked that LNT treatment led to a significant downregulation of the proliferation marker (ki67) in tumor tissues (Fig. 1D). These data validate the tumor-suppressing efficacy of LNT in NB.

### LNT inhibits NB cell growth, migration, and invasion

**Fig. 2:**
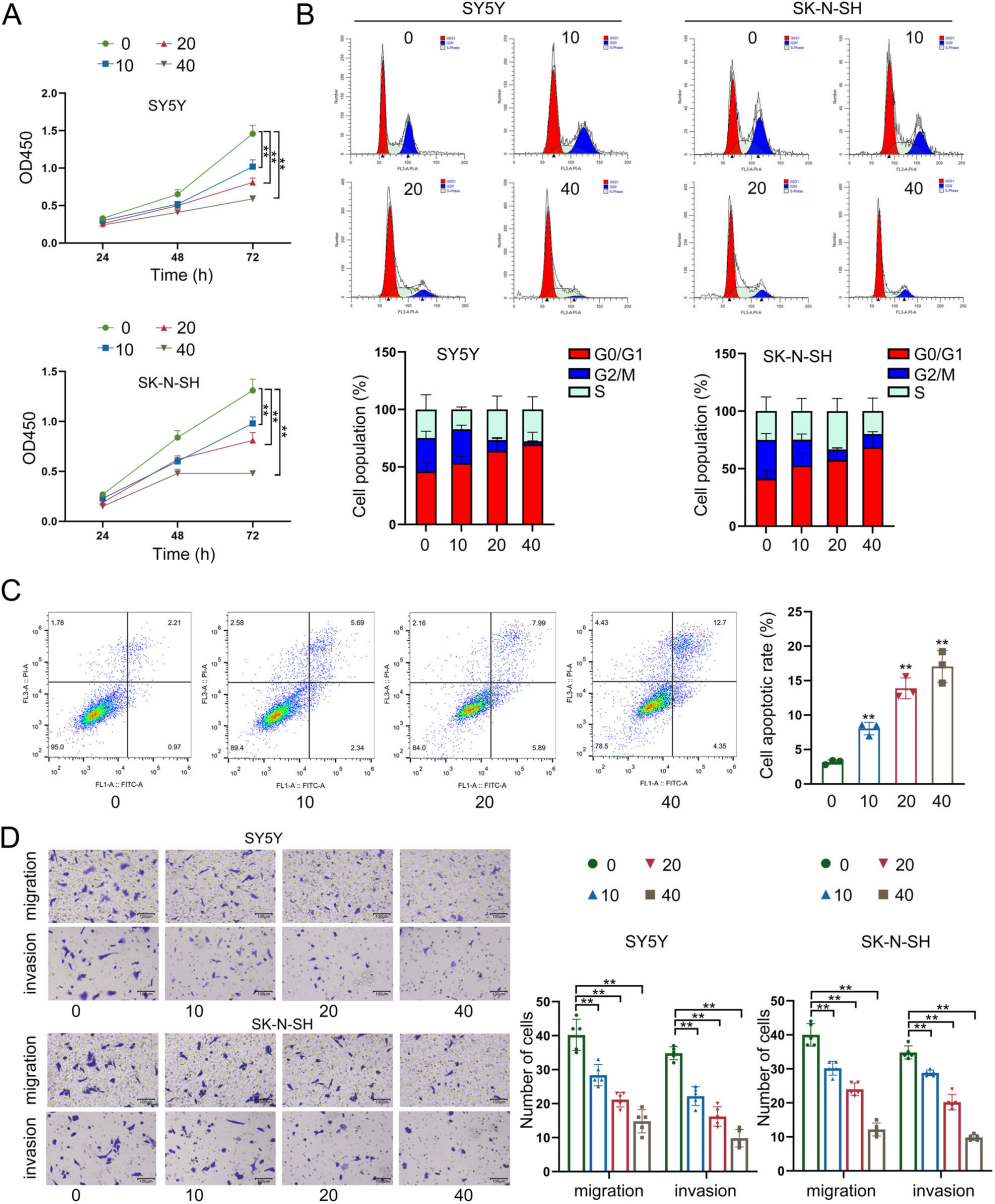
LNT inhibits NB cell growth, migration, and invasion. **A** The viability of NB cells was measured by CCK-8 assay after being treated with the increased dose of LNT (0, 10, 20, 40 μg/ml). **B** Cell cycle progress was monitored by flow cytometry in NB cells treated with different doses of LNT. **C** The apoptosis rate of NB cells treated with four different doses of LNT was measured by flow cytometry. **D** The migrating and invasive abilities of NB cells were detected by Transwell assays after being treated with four different doses of LNT. **P < 0.01, n.s.: not significant.

The in vitro experiments were designed and performed in NB cells treated with increasing dose of LNT (0, 10, 20, 40 μg/ml) to identify the functional effects of LNT. Through CCK-8 detection, the viability of NB cells was gradually decreased with the increasing dose of LNT (Fig. 2A). Cell cycle distribution was then monitored and the results indicated that NB cell cycle was stagnated at G0/G1 phase after being treated with LNT (Fig. 2B). In addition, the apoptosis rate of NB cells was obviously increased by the incremental dose of LNT (Fig. 2C). And beyond that, the migrating and invasive abilities of NB cells were gradually weakened by the incremental dose of LNT (Fig. 2D). According to the above data, LNT treatment is a suppressing factor for NB cell growth, migration, and invasion.

### LNT results in a significant downregulation of FOS expression

**Fig. 3:**
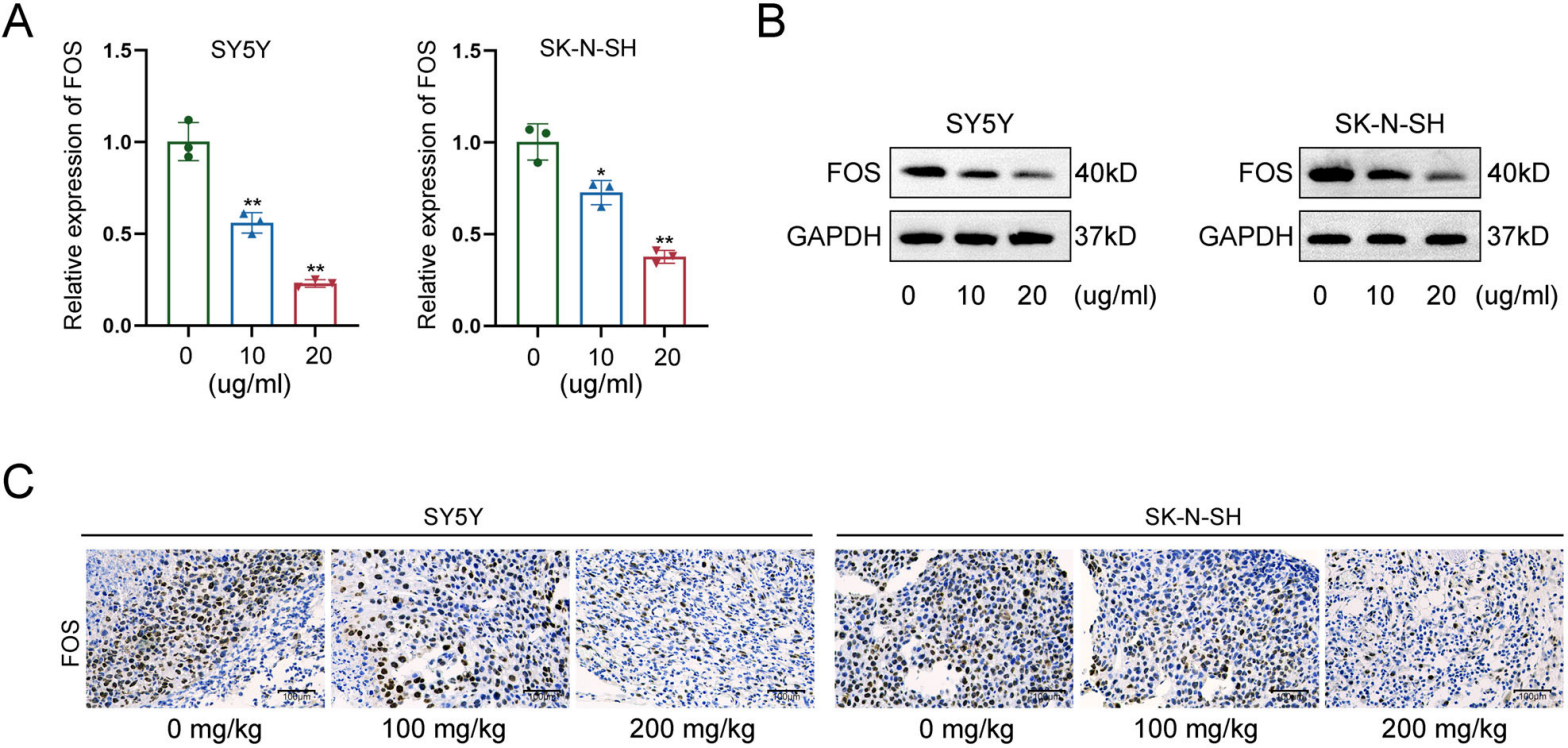
LNT results in a significant downregulation of FOS expression. **A** RNA expression of FOS was measured by RT-qPCR in NB cells treated with LNT. **B** The protein level of FOS measured by western blot in NB cells treated with LNT. **C** The positive expression of FOS was detected in tumor tissues obtained from tumor-bearing mice treated with different doses of LNT. *P < 0.05, **P < 0.01.

The subsequent investigation focused on the molecular mechanism regulated by LNT in NB cells. FOS gene has been recognized as an oncogene in NB [12, 13]; moreover, it can be modulated in cancer cells treated with anti-cancer drugs [14, 15]. The current study made an exploration on the potential involvement of FOS in LNT-mediated functional changes of NB cells. After RT-qPCR analysis, RNA expression of FOS was significantly reduced by LNT treatment (Fig. 3A). Consistently, the protein level of FOS was also changed in a decreasing tendency after LNT treatment (Fig. 3B). Moreover, tumor-bearing mice were treated with different dose of LNT for further detection. In consistent with the in vitro results, tumor growth was suppressed more efficient in model mice treated with higher dose of LNT, as reflected by the tumor size (Figure S1A), volume (Figure S1B), and weight (Figure S1C). The positive expression of FOS protein was then detected in tumors obtained from tumor-bearing mice treated with different doses of LNT. Likewise, FOS expression was decreased more in tumor tissues obtained from model mice treated with higher dose of LNT (Fig. 3C). Based on above all, we summarize that LNT can downregulate FOS expression in NB.

### Silencing of FOS exerts suppressing functions in NB cell growth, migration, and invasion

**Fig. 4:**
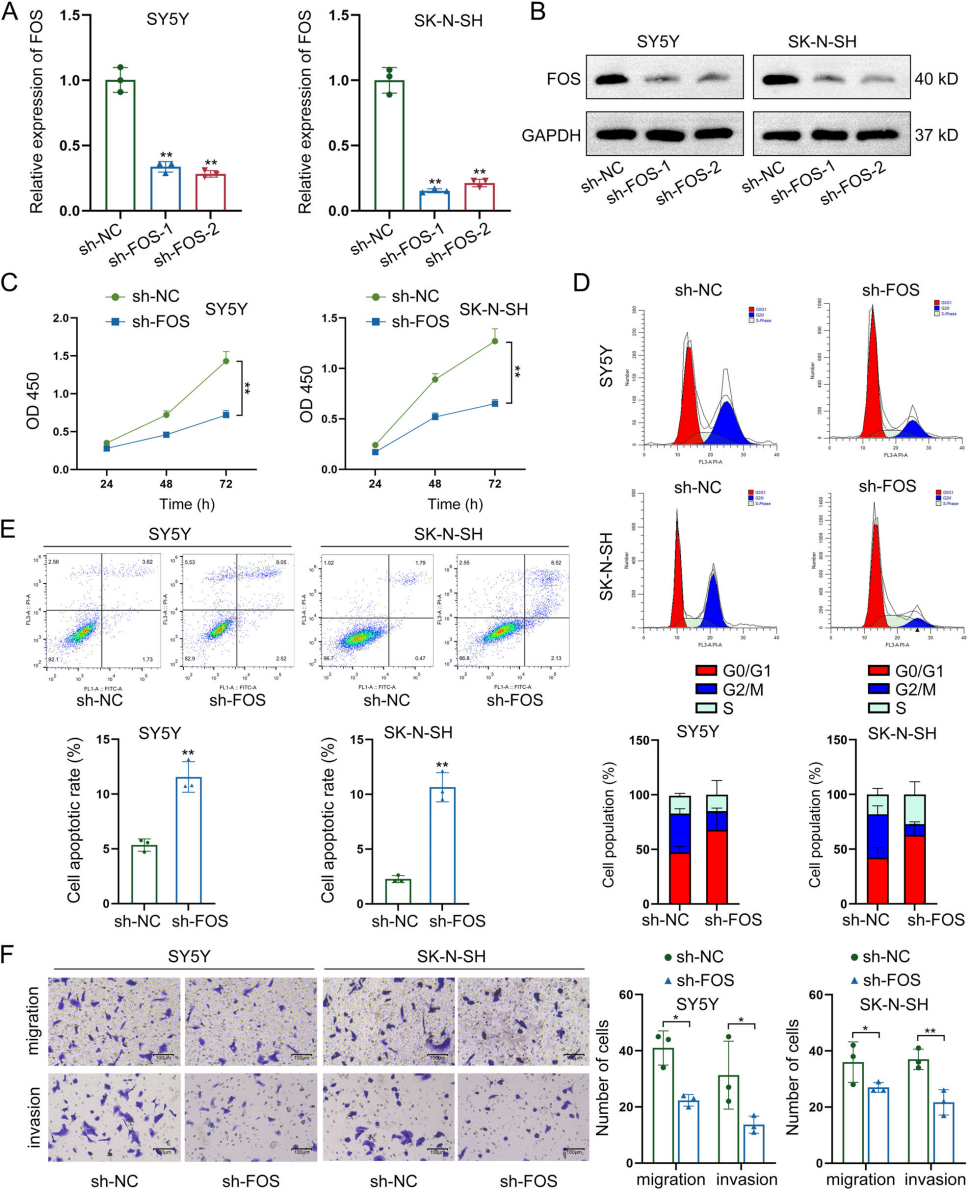
Silencing of FOS exerts suppressing functions in NB cell growth, migration, and invasion. **A**, **B** FOS expression was silenced through specific shRNAs transfection, and the effective knockdown of FOS expression was measured by RT-qPCR and western blot. **C** The viability of NB cells was evaluated by CCK-8 assay after FOS knockdown. **D** The cell cycle distribution was detected by flow cytometry after knockdown of FOS in NB cells. **E** The apoptotic condition of NB cells was assessed by flow cytometry after knockdown of FOS. **F** The migrating and invasive levels of NB cells were measured by Transwell assays after FOS knockdown. *P < 0.05, **P < 0.01.

Before identification of LNT-mediated regulatory mechanism of FOS, we silenced FOS expression through specific shRNAs transfection for loss-of-function assays. At first, the effective knockdown of FOS expression was measured by RT-qPCR and western blot (Fig. 4A, B). After FOS knockdown, the viability of NB cells presented a significant reduction, as measured by CCK-8 assay (Fig. 4C). Meanwhile, the cell cycle was arrested at G0/G1 phase after knockdown of FOS in NB cells (Fig. 4D). Moreover, FOS knockdown led to the acceleration of NB cell apoptosis (Fig. 4E). Finally, the FOS-silenced NB cells presented lower migrating and invasive levels (Fig. 4F). To make further validation, FOS was knocked out from two NB cells for same functional assays. Knockout of FOS had high efficiency in suppressing NB cell proliferation (Figure S2A), inducing cell cycle arrest (Figure S2B), increasing apoptosis rate (Figure S2C), and inhibiting migration and invasion (Figure S2D). Therefore, we confirm that FOS promotes NB cell growth, migration, and invasion.

### FOS overexpression recovers NB cells from LNT-induced functional suppression

**Fig. 5:**
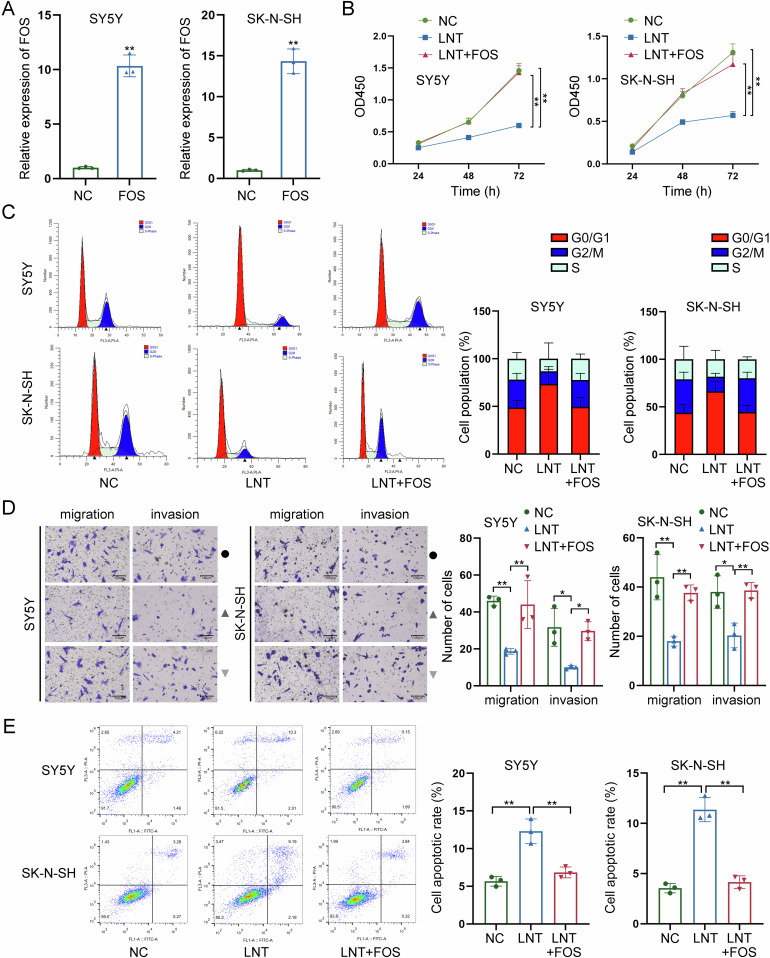
FOS overexpression recovers NB cells from LNT-induced suppression. **A** FOS was overexpressed in NB cells, and the effective overexpression was confirmed via RT-qPCR. **B** The effect of FOS overexpression on LNT-induced suppression on NB cell viability was detected by CCK-8 assay. **C** The cell cycle distribution was analyzed by flow cytometry in LNT-treated NB cells after overexpression of FOS. **D** The migration and invasion of LNT-treated NB cells were detected by Transwell assays after FOS overexpression. **E** The apoptotic condition of LNT-treated NB cells was detected by flow cytometry after overexpression of FOS. *P < 0.05, **P < 0.01.

Rescue assays were carried out to further verify the role of LNT-mediated FOS downregulation in NB. Before that, we enhanced FOS expression and confirmed the effective overexpression via RT-qPCR (Fig. 5A). Functionally, overexpression of FOS had a significant effect on strengthening the viability of NB cells decreased by LNT treatment, according to the CCK-8 detection (Fig. 5B). Next, through flow cytometry analyses, FOS overexpression was proven to effectively reverse LNT-induced cell cycle arrest (Fig. 5C). Additionally, LNT-mediated suppression on both migration and invasion were recovered after overexpression of FOS (Fig. 5D). FOS overexpression could reverse LNT-induced acceleration of apoptosis (Fig. 5E). These data further validate that LNT suppresses NB progression via downregulating FOS.

### FOS interacts with JUND to upregulate VRK1

**Fig. 6:**
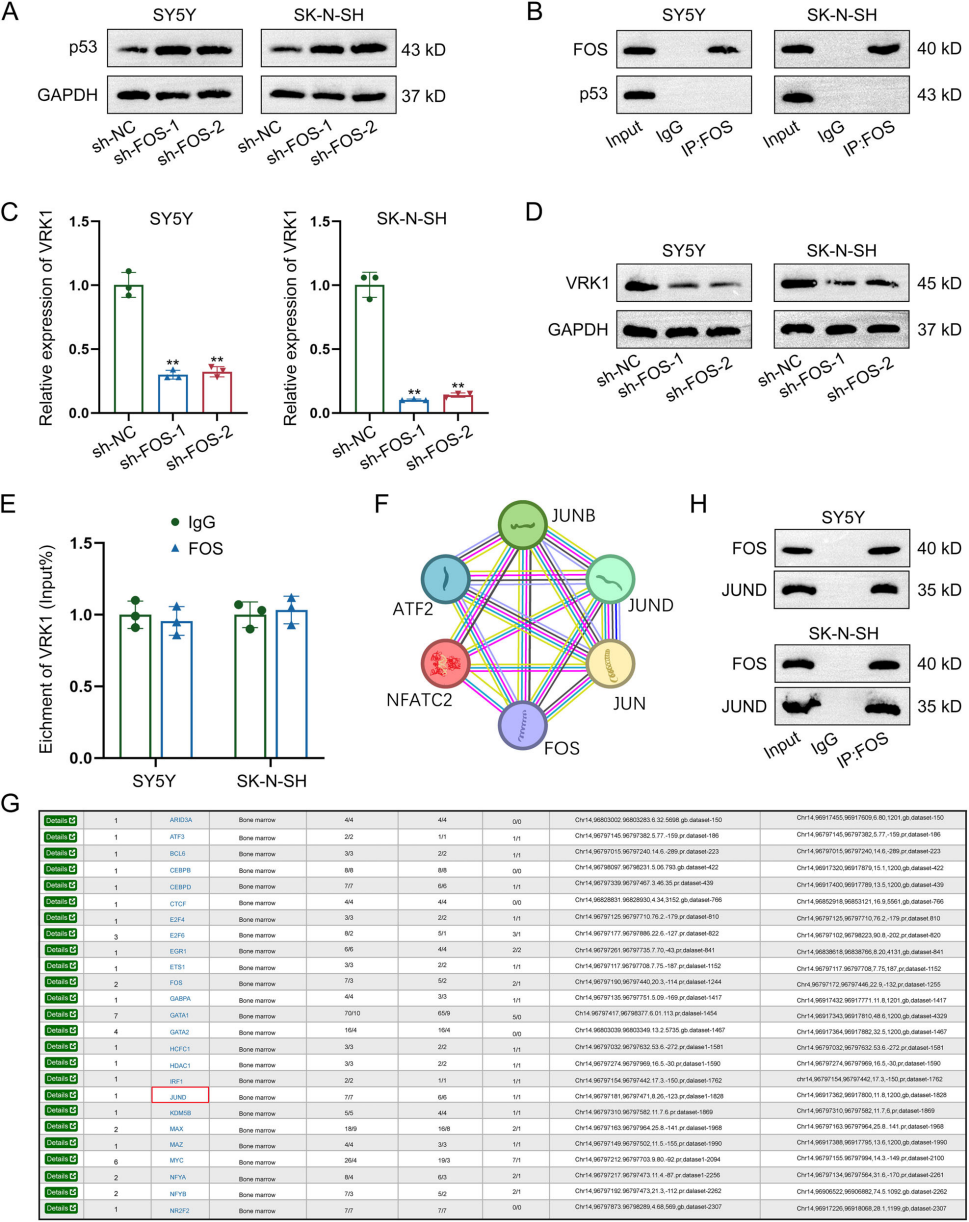
FOS interacts with JUND to upregulate VRK1. **A** The protein level of p53 was measured in NB cells with FOS knockdown by western blot. **B** The interaction between FOS and p53 was validated by Co-IP assay. **C**, **D** The mRNA and protein levels of VRK1 were separately measured in FOS-silenced NB cells by RT-qPCR and western blot. **E** The potential interaction between FOS and VRK1 was detected by RIP assay. **F** The FOS-interacting proteins were predicted by screening on STRING. **G** hTFtarget predicted that JUND was an upstream transcription factor of VRK1. **H** The direct interaction between FOS and JUND was demonstrated by co-IP assay. **P < 0.01.

Studies have indicated that p53-MDM2 pathway is a crucial factor that can regulate cell apoptosis and cell cycle progress [18]. Evidence has revealed the suppressing effect of FOS on p53 [19]. However, the specific mechanism of FOS-mediated p53 downregulation in NB progression remains unclear. Here, we measured p53 protein level in NB cells with FOS knockdown and found the increasing tendency (Fig. 6A). As detected by co-IP assay, there was no direct interaction between FOS and p53 (Fig. 6B). Since VRK1 can regulate p53 expression to affect DNA injury and it can also modulate cell cycle distribution in NB [27, 28], we next analyzed the correlation between FOS and VRK1. According to data obtained from RT-qPCR and western blot, both mRNA and protein levels of VRK1 were reduced by FOS knockdown, suggesting the positive regulation of FOS on VRK1 expression (Fig. 6C, D). As demonstrated by RIP assay, FOS could not directly interact with VRK1 in NB cells (Fig. 6E). In subsequence, the FOS-interacting proteins were predicted by screening on STRING (https://cn.string-db.org/). As shown in Fig. 6F, FOS could interact with multiple transcription factors. Hence, we hypothesized that FOS might interact with a certain transcription factor to regulate the transcription activity of VRK1. To validate our hypothesis, we applied the bioinformatics analysis tool hTFtarget (http://bioinfo.life.hust.edu.cn/hTFtarget#!/) to predict the potential upstream transcription factor of VRK1. It was found that JUND was the potential transcription regulator of VRK1 (Fig. 6G). Hereto, we could suppose that FOS might interact with JUND to regulate VRK1 transcription. Further, co-IP data indicated that FOS had a direct interaction with JUND (Fig. 6H). Based on all above results, we suppose that FOS may regulate VRK1 transcription via JUND.

### FOS transcriptionally activates VRK1 through JUND

**Fig. 7:**
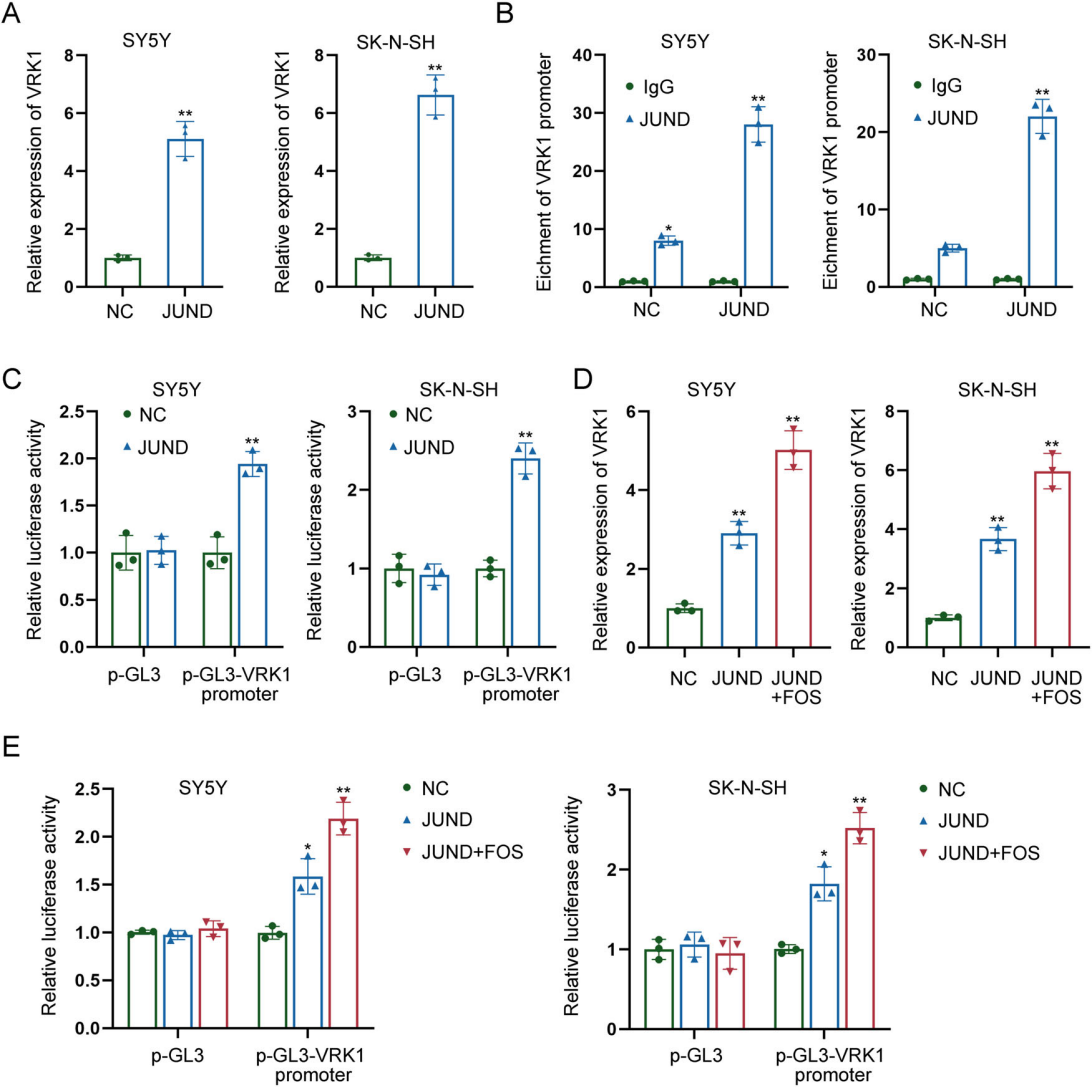
FOS transcriptionally activates VRK1 through JUND. **A** The expression level of VRK1 was detected by RT-qPCR in JUND-overexpressed NB cells. **B** ChIP assay indicated the strong affinity of JUND to VRK1 promoter. **C** The promoter activity of VRK1 was measured in NB cells with JUND overexpression by luciferase reporter assay. **D** VRK1 expression was measured by RT-qPCR in NB cells transfected with JUND overexpression vector or both JUND and FOS overexpression vectors. **E** The promoter activity of VRK1 was measured by luciferase reporter assay in NB cells transfected with JUND overexpression vector or both JUND and FOS overexpression vectors. *P < 0.05, **P < 0.01.

In subsequence, we focused on whether FOS affected VRK1 transcription via JUND. RT-qPCR analysis revealed the positive effects of JUND on VRK1 expression, as evidenced by the enhanced expression level of VRK1 in JUND-overexpressed NB cells (Fig. 7A). Further ChIP assay indicated that JUND had strong affinity to VRK1 promoter (Fig. 7B). According to the results of luciferase reporter assay, the promoter activity of VRK1 was enhanced a lot by the overexpression of JUND (Fig. 7C). Moreover, VRK1 expression was upregulated by JUND overexpression, while it was upregulated most by co-overexpression of JUND and FOS (Fig. 7D). Similarly, co-overexpression of JUND and FOS had a higher efficiency than overexpression of JUND in enhancing the promoter activity of VRK1 (Fig. 7E). The aforementioned results reveal that FOS activates VRK1 transcription via JUND.

### VRK1 induces the downregulation of p53 via phosphorylation regulation

**Fig. 8:**
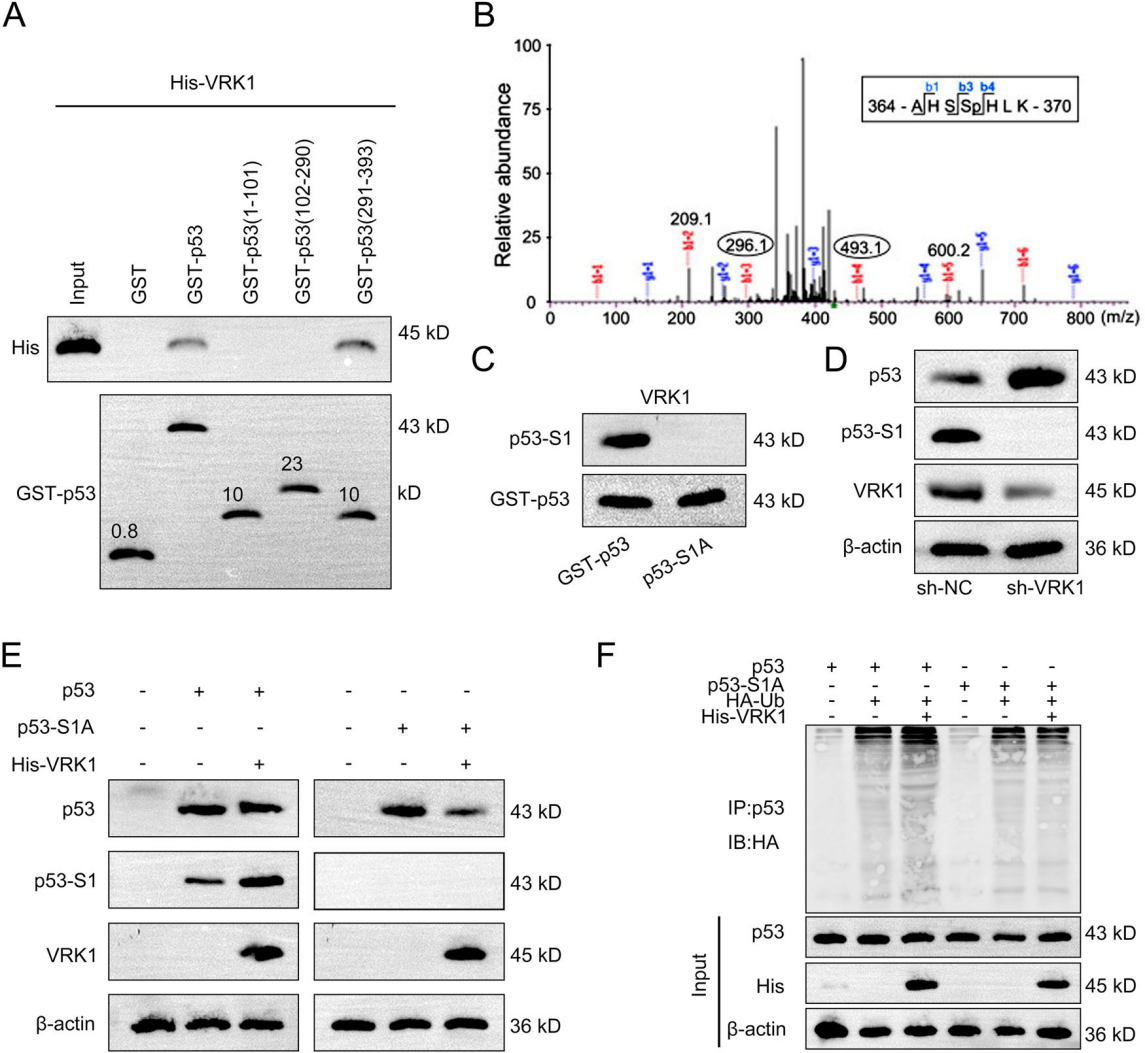
VRK1 induces the downregulation of p53 via phosphorylation regulation. **A** The specific interaction region of VRK1 with p53 was detected by GST-pulldown assay followed by western blot analysis. **B** The phosphorylation site of p53 (named S1) was analyzed by the mass spectrometry. **C** The interaction between VRK1 and S1 of p53 was detected by western blot analysis before or after mutation of S1. **D** The protein level of p53 and corresponding phosphorylation level of p53 were measured by western blot in NB cells with VRK1 knockdown. **E** The interaction between VRK1 and p53 or S1 was detected by western blot after mutation of S1. **F** The ubiquitination level of p53 was also detected before or after mutation of S1.

Considering the regulating effects of FOS on VRK1 and p53, we then explored the potential correlation between VRK1 and p53. Firstly, we conducted GST-pulldown assay and applied western blot to analyze the results. As the results, VRK1 could directly interact with site 291–393 of p53 protein (Fig. 8A). Furthermore, the phosphorylation site of p53 (named S1) was analyzed by the mass spectrometry (Fig. 8B). S1 was then mutated for further western blot analysis, and the results indicated that VRK1 could interact with p53 at S1, while the interaction was abolished after mutation of S1 (Fig. 8C). We then detected the effects of VRK1 on the levels of p53 protein and its phosphorylation level. According to the results shown in Fig. 8D, VRK1 knockdown led to a significant increase of p53 protein level but an obvious decrease of p53 phosphorylation level. Meanwhile, mutation of S1 blocked the interaction between VRK1 and the S1 of p53 and weakened the interaction between VRK1 and p53 (Fig. 8E). The ubiquitination level of p53 was also detected before or after mutation of S1. It was found that VRK1 promoted the phosphorylation of p53 but suppressed the ubiquitination of it (Fig. 8F). Taken together, VRK1 downregulates p53 protein by promoting its phosphorylation to inhibit ubiquitination.

## Discussion

Our current study revealed the anti-tumor efficacy of LNT in NB. Functionally, LNT treatment led to the inhibition of NB cell growth, migration, and invasion. Mechanistically, we uncovered that LNT induced the downregulation of FOS to inactivate VRK1 transcription. Furthermore, we found that VRK1 downregulated p53 protein by promoting its phosphorylation to inhibit ubiquitination.

NB has strong proliferative ability, high resistance to apoptosis. Although standard therapies, including chemotherapy, surgery, radiotherapy, and immunotherapy have been applied to improve the treatment of NB patients [29], the prognosis remains unfavorable due to the resistance to conventional therapy [30]. Therefore, it is necessary to identify novel therapeutic targets or drugs. LNT has been used to be an adjuvant drug for anti-cancer therapy in China [8]. Our current study firstly unveiled the anti-tumor potential of LNT in NB. Through in vivo investigations, we demonstrated that LNT treatment could suppress tumor growth in model mice, as shown by the decreased tumor size, weight, and volume. Moreover, in vitro experiments were performed in NB cells treated with increasing dose of LNT and the results indicated the role of LNT in inhibiting malignant processes of NB cells. With the increasing dose of LNT, the viability of NB cells was gradually reduced, the cell cycle was stagnated at G0/G1 phase, the apoptosis rate was increased, and the migrating and invasive abilities of NB cells were weakened. Hence, we confirmed that LNT treatment led to suppression on NB cell growth, migration, and invasion.

Our investigation also focused on the molecular mechanism regulated by LNT treatment in NB cells. FOS is a proto-oncogene and it can be activated to exert tumor-promoting function in various human cancers through regulating cell cycle progress and apoptosis [31, 32]. The upregulation of FOS has been detected in NB cells [33]. Moreover, the oncogenic functions of FOS in NB can be suppressed by DNMT3B isoform DNMT3B7 [34]. To date, the specific role of FOS in NB progression and whether it can be regulated by LNT remain unclear. Here, we measured FOS expression in NB cells or model mice after LNT treatment. And FOS expression was obviously downregulated by LNT treatment. Through functional experiments, we identified that knockdown of FOS could effectively reduce the viability of NB cells, induced cell cycle arrest, accelerated apoptosis, and restrained migration and invasion. Subsequently, rescue assays revealed that overexpression of FOS recovered the growth, invasion, and migration of NB cells suppressed by LNT. According to all these results, we summarized that LNT downregulated FOS expression to exert suppressing functions in NB cell growth, migration, and invasion.

The next step was to identify FOS-mediated regulatory mechanism in NB. p53 has been acknowledged as a tumor suppressor in multiple human cancers due to its crucial role in modulating cell cycle arrest and apoptosis [35, 36]. The present study validated that p53 protein level could be increased in NB cells after FOS knockdown. However, the co-IP data indicated that there was no direct interaction between FOS and p53. Since VRK1 could regulate cell cycle distribution in NB and it could also modulate p53 expression to affect DNA injury [27, 28], we analyzed the correlation between FOS and VRK1. Through mechanism experiments and bioinformatics analyses, this study revealed that FOS could interact and cooperate with JUND to activate VRK1 transcription. Furthermore, this study explored the correlation between VRK1 and p53. According to results of GST-pulldown assay and mass spectrometry analysis, VRK1 induced the phosphorylation of p53 protein at site 291–393. Furthermore, we demonstrated that VRK1 enhanced the ubiquitination level of p53 at the phosphorylation site 1. All these mechanism investigations indicated that VRK1 downregulated p53 protein by promoting its phosphorylation to inhibit ubiquitination.

To conclude, our research findings unveiled the anti-tumor efficacy of LNT in NB and discovered its downstream molecular mechanism. LNT could downregulate FOS that interacted and cooperated with JUND to transcriptionally activate VRK1. LNT could further suppress the phosphorylation of p53 and enhanced the protein stability of p53 by downregulating VRK1. Our findings may help to find new therapeutic targets and effective drugs for NB patients. Several limitations of the current study need to be broken. On one hand, the current study only focused on the regulatory mechanism by which LNT suppressed FOS-mediated transcription activation of VRK1 to stabilize p53 protein. Therefore, we will explore more potential mechanisms regulated by LNT in NB progression in our future study. On the other hand, it is unknown whether different MW marker and quality of LNT will change the therapeutic effect and mechanism of LNT, which will be further explored in our future study.

## Materials and methods

### Cell culture and treatment

Two NB cell lines (SK-N-SH and SY5Y) were obtained from the Type Culture Collection of the Chinese Academy of Sciences (Shanghai, China). SK-N-SH cell line was cultured in DMEM with supplementation of 10% FBS and 1% penicillin-streptomycin. SY5Y cell line was cultured in a 1:1 mixture of MEM and F12 Medium with additional 10% FBS, 1% penicillin-streptomycin solution, 1% Gluta-max, 1% Sodium pyruvate, and 1% NEAA. All mediums and solutions used for cell culture were purchased from Gibco (CA, USA). Cells were maintained at 37 °C in a humidified incubator with 5% CO_2_.

To identify the anti-tumor efficacy of LNT, LNT purchased from MedChemExpress (NJ, USA) was used to treat NB cells at different doses (0, 10, 20, 40 μg/ml). The LNT extracted from the Shiitake mushroom (Lentinula edodes) with the hot water extraction method, as previously described [37].

### Cell transfection

Short hairpin RNAs specifically targeting FOS (sh-FOS-1/2) and VRK1 (sh-VRK1) and their corresponding negative control shRNA (sh-NC) were synthesized by Shanghai GeneChem Co., Ltd. pc-DNA3.1 vector containing the whole length of FOS or JUND (named FOS or JUND) was separately generated for overexpressing FOS or JUND, and the empty pc-DNA3.1 vector was taken as the negative control (NC). Lipofectamine® 2000 (Invitrogen, CA, USA) was applied to transfect the recombinants into NB cells.

### Reverse transcription-quantitative PCR (RT-qPCR)

Total RNA extracted by TRIzol® reagent (Invitrogen) was then subjected to reverse transcription to generate cDNA by using the PrimeScript RT Master Mix (Takara Bio, Inc.). The amplification of cDNA was finished in an ABI PRISM 7900 Real-Time system (Applied Biosystems, Foster City, CA, USA) by using a SYBR PrimeScript RT-PCR kit (Takara Bio, Inc.). Through normalizing to GAPDH, relative mRNA level was determined with 2^−ΔΔCT^ calculation method.

### Western blot

The RIPA buffer (Auragene Bioscience Co.) was used to extract total proteins from indicated NB cells. The extracted proteins were then separated by 10% SDS-PAGE (Bio-Rad Laboratories, Inc.), followed by transferring onto PVDF membranes (Millipore, Bedford, MA, USA). Next, the membranes were incubated with primary antibodies against GAPDH (1:1000), β-actin (1:1000), FOS (1:1000), p53 (1:1000), VRK1 (1:1000), and JUND (1:1000) at 4 °C overnight. After washing four times in TBST, the membranes were incubated further with the HRP-conjugated secondary antibodies (1:2000). All abovementioned antibodies were obtained from Abcam (Cambridge, CA, USA). After being visualized on an ECL detection system (Beyotime Institute of Biotechnology), the density of each protein band was analyzed with the ImageJ software (NIH, Bethesda, MD, USA).

### Cell counting kit 8 (CCK-8) assay

The viability of NB cells after indicated treatments or transfections was assessed by performing CCK-8 assay. Briefly, 5 × 10^3^ NB cells were seeded into each well of 96-well plates. After being incubated for 24, 48, or 72 h, each well was added with 10 μl CCK-8 reagent and was incubated for another 3 h. Finally, a microplate reader was applied to measure the optical density (OD) value at a wavelength of 450 nm.

### Flow cytometry analysis

The apoptotic conditions or cell cycle distribution were detected in accordance with procedures mentioned in previous studies [38, 39]. Experiments were carried out using FACSCalibur flow cytometer (BD Biosciences, USA), and the data analyses were finished by using FlowJo 7.6 software.

### Transwell assays

To evaluate the migrating and invasive abilities of NB cells, 4 × 10^4^ NB cells were seeded into the upper chamber coated with or without Matrigel (BD Bioscience) of a Transwell insert with 8-mm pore size (Corning Inc., NY, USA) containing serum-free medium. The medium in the lower chamber was supplemented with 20% FBS to make a chemoattractant. Forty-eight hours later, the medium was discarded, cells migrated or invaded into the lower chamber were treated with methanol for fixation and then with 0.1% crystal violet for staining. Results were observed under an optical microscope.

### In vivo experiments

The animal study was approved by the Institutional Animal Care and Use Committee of Guangzhou Women and Children’s Medical Center, with the ethics approval number 2023031010493265. Forty BALB/c nude mice (4–6 weeks) raised at the Animal Experimental Center of Guangdong Medical Laboratory Animal Center were used in this study. At first, tumor-bearing mice were established. To identify the effect of dosage changes of LNT on tumor growth, tumor-bearing mice were established by subcutaneously injecting 1 × 10^7^ SY5Y cells into the right flank of each nude mouse. After tumor formation, all tumor-bearing mice were divided into three groups: SY5Y (0 mg/kg), SY5Y (100 mg/kg), and SY5Y (200 mg/kg). Each tumor-bearing mouse in three groups was injected intraperitoneally with 0, 100, or 200 mg/kg/daily LNT for 5 days, respectively. Similarly, 1 × 10^7^ SK-N-SH cells were subcutaneously injected into the right flank of each nude mouse to establish tumor-bearing mice. After tumor formation, all tumor-bearing mice were divided into three groups: SK-N-SH (0 mg/kg), SK-N-SH (100 mg/kg), and SK-N-SH (200 mg/kg). Each tumor-bearing mouse in three groups was injected intraperitoneally with 0, 100, or 200 mg/kg/daily LNT for 5 days, respectively. To identify the tumor growth condition in mice treated with or without LNT, 1 × 10^7^ SY5Y cells were subcutaneously injected into the right flank of each nude mouse to establish tumor-bearing mice. All mice were divided into two groups: SY5Y (Control) and SY5Y (LNT). Each tumor-bearing mouse in Control group was injected intraperitoneally with PBS, while each in LNT group was injected intraperitoneally with 200 mg/kg/daily LNT for 5 days. Similarly, 1 × 10^7^ SK-N-SH cells were subcutaneously injected into the right flank of each nude mouse to establish tumor-bearing mice. All mice were divided into two groups: SK-N-SH (Control) and SK-N-SH (LNT). After tumor formation, each mouse in LNT group was injected intraperitoneally with 200 mg/kg LNT or equal amount of PBS. Tumor volume was monitored and recorded every three days from 7th day after injection. All mice were sacrificed at 28th day after injection for tumor resection. The weight of resected tumors in each group was measured. Tissues were isolated from tumors in each different group for immunohistochemical (IHC) staining by using anti-ki67 antibody (Abcam).

### Establishment of FOS-knockout cell lines

To further determine the role of FOS in NB, CRISPR/Cas9 single-vector lentivirus LV-FOS-sgRNA synthesized by Biocytogen Pharmaceuticals (Beijing) Co., Ltd was applied to introduce Cas9 protein and sgRNA sequence into two NB cells to knock out FOS gene.

### Co-immunoprecipitation (co-IP)

Cellular co-IP assay was conducted to demonstrate protein–protein interaction by referring to the procedures mentioned in a previous study [40]. Briefly, the lysates of NB cells were obtained by using RIPA lysis buffer (Beyotime) with supplementation of a protease inhibitor cocktail (Roche). Next, 30 μL of protein G beads (Life Technologies) was used to preclear a total of 2 mg whole cell lysates. The lysates were then incubated with 2 μg of IgG control or anti-FOS for 2 h on a rocking platform. Finally, the immunoprecipitates were centrifuged and analyzed by western blot.

### Chromatin immunoprecipitation (ChIP) assay

The binding affinity of JUND to VRK1 promoter was evaluated by ChIP assay as mentioned previously [41]. In brief, the cross-link of cells was conducted by using 1% formaldehyde to treat cells for 10 min at 37 °C. Next, 2.5 M glycine was used to quench cell samples at room temperature for 5 min. After ultrasound rupture of chromatin, anti-JUND antibody (1:200 dilution; Santa Cruz) and negative control anti-IgG (1 µg/ml dilution; Abcam) were used to obtain DNA immunoprecipitates from cell lysates. The precipitates of crosslinked protein-DNA complexes were collected, and then the DNA was purified for qPCR analysis.

### Luciferase reporter assay

According to the procedures described in a previous study [42], luciferase reporter assay was performed to verify the effect of JUND on the promoter activity of VRK1. Briefly, the promoter region of VRK1 was subcloned into a pGL3-basic vector (Promega Corporation) to construct pGL3-VRK1 promoter vector. The empty pGL3 vector was set as the negative control. Next, NB cells were co-transfected with two reporter vectors (pGL3-VRK1 promoter and pGL3) and JUND overexpression vector or NC using Lipofectamine 2000. Forty-eight hours later, a Dual-Luciferase Reporter assay system (Promega) was applied for the measurement of relative luciferase activity.

### GST-pulldown assay

GST pulldown assay was performed to validate the direct interaction between proteins, according the procedures described in a previous study [43]. Briefly, the GST-p53, GST-p53 (1–101), GST-p53 (102–290), GST-p53 (291–393), and His-VRK1 were cloned and synthesized. Next, they were inserted into pGEX-6P-1. After that, protein samples containing 500 μg of GST (control group) or equal amounts of experimental groups, including GST-p53, GST-p53 (1–101), GST-p53 (102–290), GST-p53 (291–393) were mixed with glutathione-agarose resin for 3 h. Next, all groups were mixed with 500 μg of His-VRK1 protein overnight. The next day, all groups were centrifuged and added with protein loading buffer for incubation in a 100 °C water bath for 5 min. Finally, the GST, GST-p53, GST-p53 (1–101), GST-p53 (102–290), GST-p53 (291–393) and His-VRK1 were subjected to western blot analysis by separately using an anti-GST antibody and an anti-His antibody.

### Ubiquitination assay

NB cells were transfected with HA-Ub and p53/p53-S1A for 36 h, the ubiquitinated p53 was purified by Ni-NTA agarose beads from the cell extracts. Cells stably expressing His-VRK1 were then harvested for immunoprecipitation. Purified His-VRK1 were eluted with His-peptides (Sigma). The ubiquitinated p53 protein was incubated with purified His-VRK1 in a deubiquitination buffer (50 mM Tris-HCl pH 8.0, 50 mM NaCl, 1 mM EDTA, 10 mM DTT, 5% glycerol) at room temperature for 4 h. Finally, the results were analyzed by western blot.

### Statistical analysis

The SPSS software (22.0) was applied to do statistical analysis of all collected experimental data. All data meet normal distribution. Data were shown in graphs as the mean ± standard deviation by using GraphPad Prism 8. Comparison of two independent groups was made by using t test, while that of multiple groups was performed by using ANOVA. *P* < 0.05 was taken as the threshold of statistical significance.

## Supplementary information

supplementary information

## Data Availability

The datasets generated during and/or analyzed during the current study are available from the corresponding author on reasonable request.
