# Supplementary material for: Lentinan suppresses the progression of neuroblastoma by inhibiting FOS-mediated transcription activation of VRK1 to stabilize p53 protein
Source: Cell Death Discov. 2025 Mar 15;11:103. doi: 10.1038/s41420-025-02315-0 (PMC11910558; doi:10.1038/s41420-025-02315-0)
Supplement: Supplementary file 1 — supplementary information [file 41420_2025_2315_MOESM1_ESM.docx]

**Figure S1 LNT suppresses tumor growth in a dose-dependent manner**

**
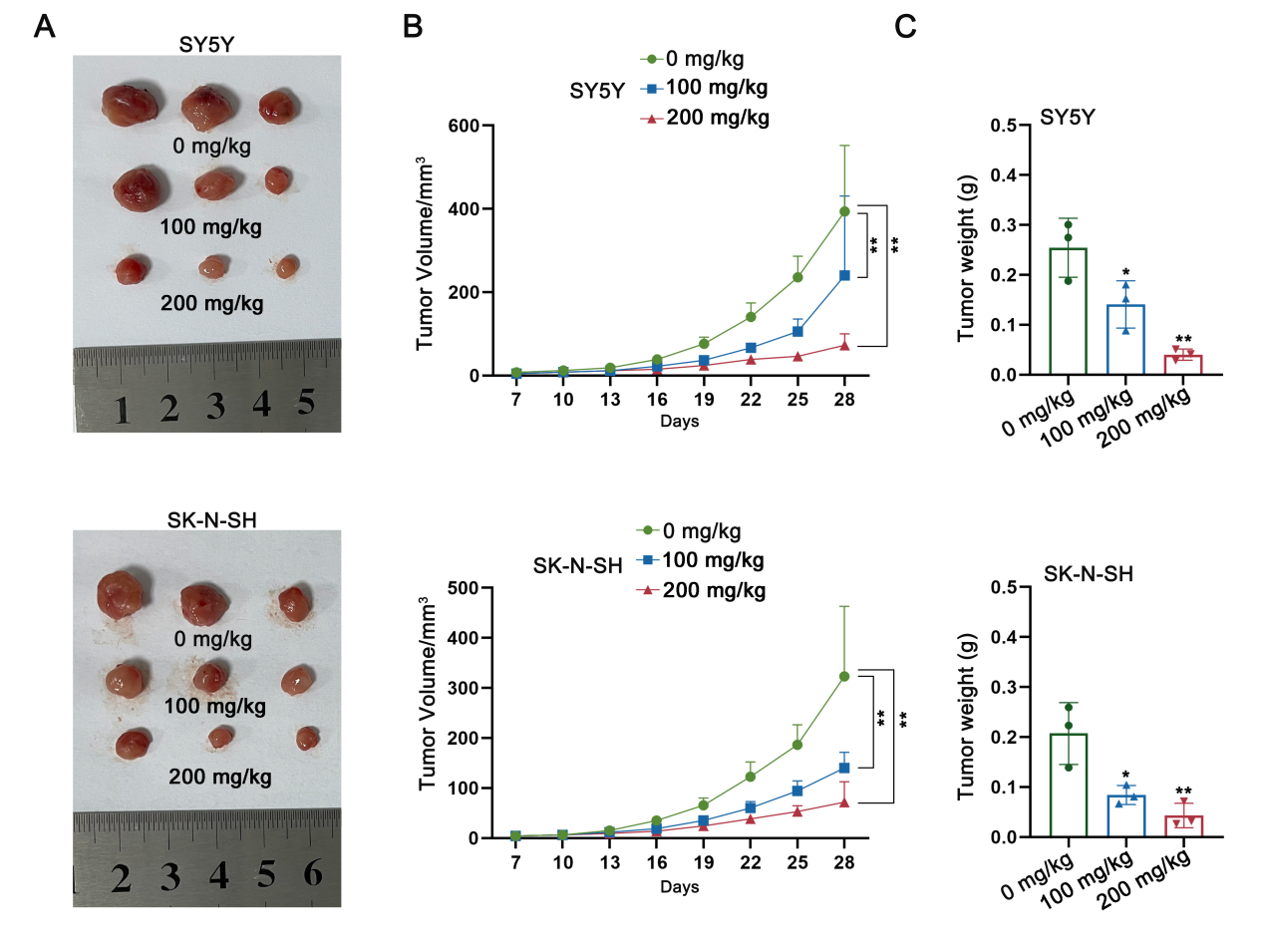
**

Tumor-bearing mice models were injected intraperitoneally with 0, 100, or 200 mg/kg/daily LNT for 5 days. **A.** Twenty-eight days later, tumors were resected and shown. **B.** Tumor volume in three different groups were measured and calculated. **C.** Tumor weight in three groups was counted and illustrated. ^*^P<0.05, ^**^P<0.01.

**Figure S2 Knockout of FOS suppresses NB cell proliferation, migration and invasion efficiently**


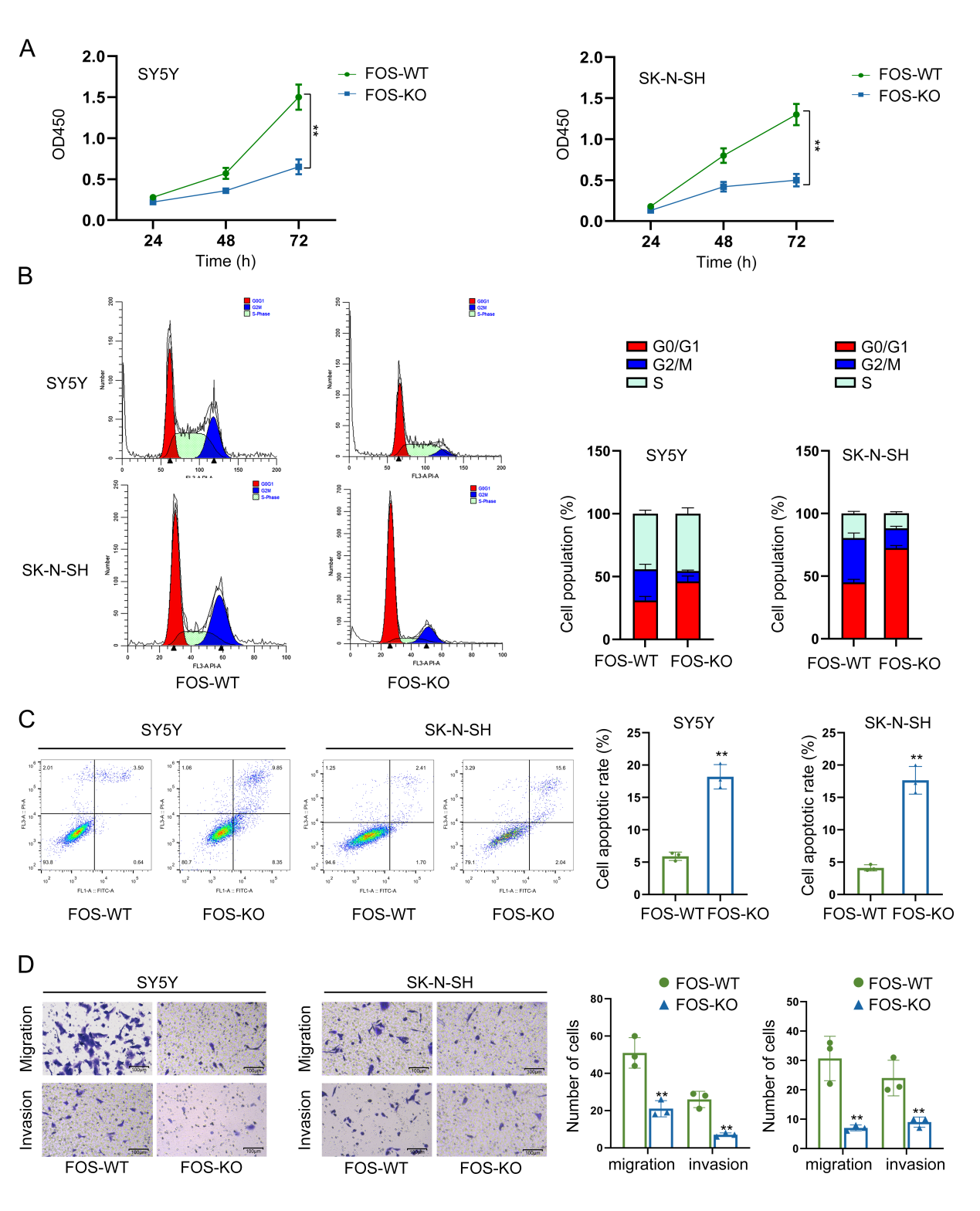

**A.** The viability of NB cells was evaluated by CCK-8 assay after FOS knockout. **B.** The cell cycle distribution was detected by flow cytometry after knockout of FOS in NB cells. **C.** The apoptotic condition of NB cells was assessed by flow cytometry after knockout of FOS. **D.** The migrating and invasive levels of NB cells were detected by Transwell assays after FOS knockout. ^**^P<0.01.

**Original Data 1**

**
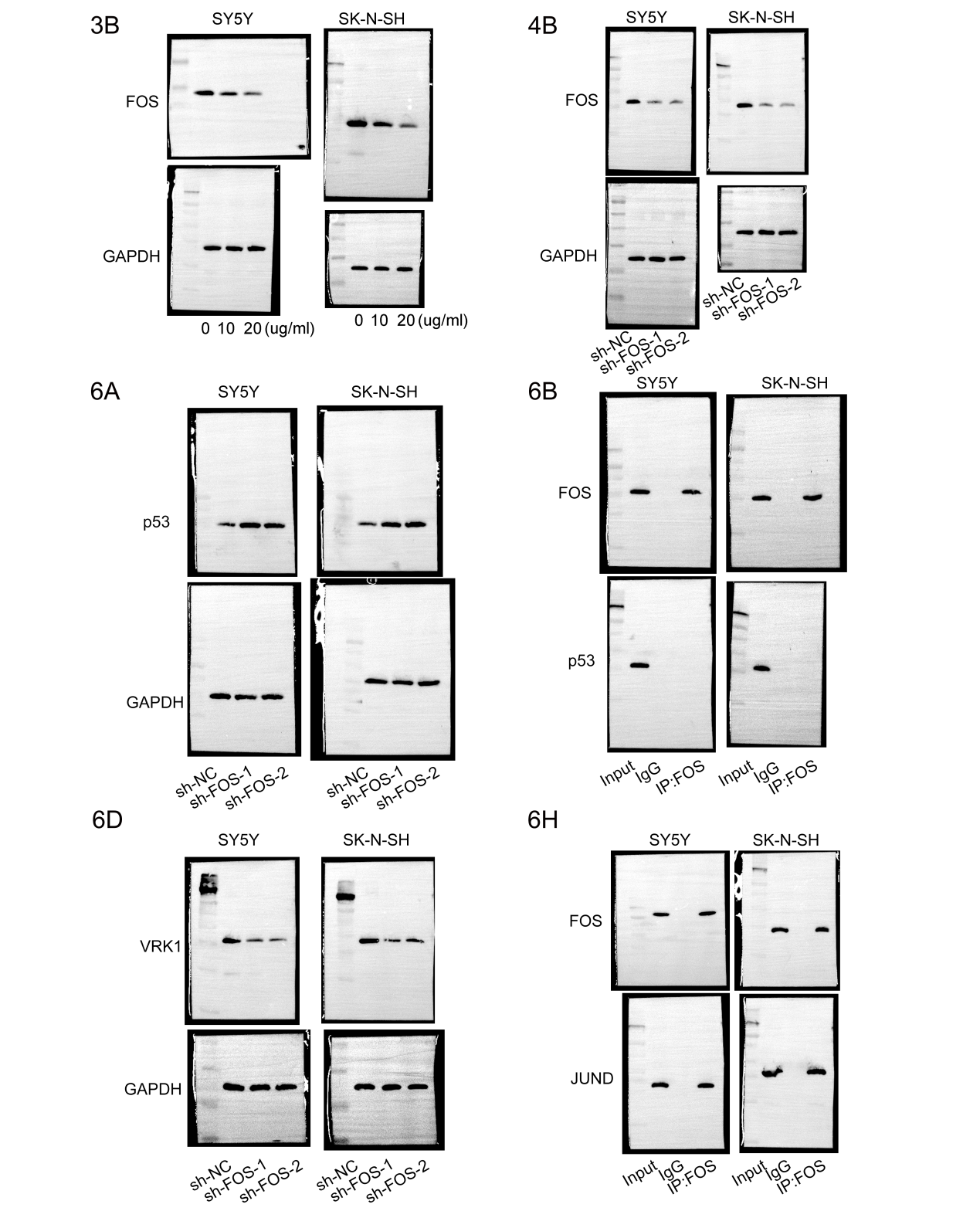
**

**Original Data 2**

**
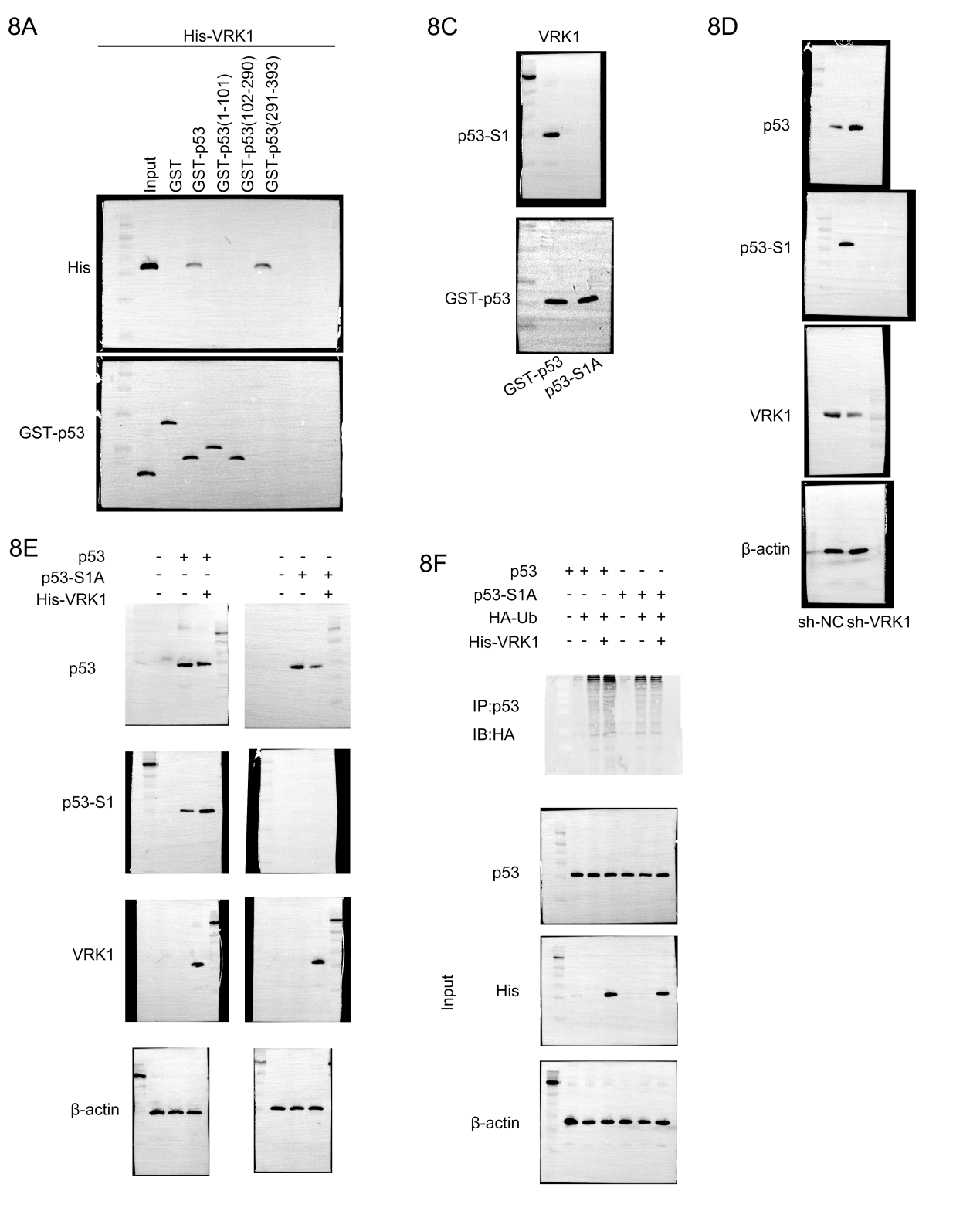
**
